# Wastewater surveillance of antibiotic resistance and class 1 integron-integrase genes: Potential impact of wastewater characteristics on genes profile

**DOI:** 10.1016/j.heliyon.2024.e29601

**Published:** 2024-04-30

**Authors:** Zahra Shamsizadeh, Mahnaz Nikaeen, Farzaneh Mohammadi, Marzieh Farhadkhani, Mehdi Mokhtari, Mohammad Hassan Ehrampoush

**Affiliations:** a Department of Environmental Health Engineering, School of Health, Larestan University of Medical Sciences, Larestan, Iran; b Environmental Science and Technology Research Center, Department of Environmental Health Engineering, School of Public Health, Shahid Sadoughi University of Medical Sciences, Yazd, Iran; c Department of Environmental Health Engineering, School of Health, Isfahan University of Medical Sciences, Isfahan, Iran; d Educational Development Center, Shahrekord University of Medical Sciences, Shahrekord, Iran

**Keywords:** Wastewater treatment plants, Antibiotic resistance genes, Mobile genetic elements, qPCR

## Abstract

Antibiotic resistance (AR) is a major global health concern, but current surveillance efforts primarily focus on healthcare settings, leaving a lack of understanding about AR across all sectors of the One Health approach. To bridge this gap, wastewater surveillance provides a cost-effective and efficient method for monitoring AR within a population. In this study, we implemented a surveillance program by monitoring the wastewater effluent from two large-scale municipal treatment plants situated in Isfahan, a central region of Iran. These treatment plants covered distinct catchment regions and served a combined population about two million of residents. Furthermore, the effect of physicochemical and microbial characteristics of wastewater effluent including biological oxygen demand (BOD), chemical oxygen demand (COD), total suspended solids (TSS), temperature, total coliforms and *Escherichia coli* concentration on the abundance of ARGs (*bla*_CTX-M_, *tet*W, *sul*1, *cml*A, and *erm*B) and class 1 integron-integrase gene (*intI*1) were investigated. *Sul*1 and *bla*_CTX-M_ were the most and least abundant ARGs in the two WWTPs, respectively. Principal Component Analysis showed that in both of the WWTPs all ARGs and *intI*1 gene abundance were positively correlated with effluent temperature, but all other effluent characteristics (BOD, COD, TSS, total coliforms and *E. coli*) showed no significant correlation with ARGs abundance. Temperature could affect the performance of conventional activated sludge process, which in turn could affect the abundance of ARGs. The results of this study suggest that other factors than BOD, COD and TSS may affect the ARGs abundance. The predicted AR could lead to development of effective interventions and policies to combat AR in the clinical settings. However, further research is needed to determine the relationship between the AR in wastewater and clinical settings as well as the effect of other influential factors on ARGs abundance.

## 1 Introduction

Over the years, antibiotics have been widely used in the prevention and treatment of diseases in both humans and animals. However, the usage of these compounds, whether appropriate or not, along with other factors, has created selective pressure leading to the emergence and spread of antibiotic-resistant bacteria (ARB) in various environments [1]. Antimicrobial resistance poses a significant challenge to multiple aspects of human life, including healthcare, food security, and the economy [1,2]. The rise in antibiotic resistance (AR) has reached alarming levels worldwide, rendering common diseases untreatable and increasing the health risks associated with antibiotic resistant pathogenic bacteria [3].

The majority of ARBs may reside within the commensal bacterial flora of healthy individuals, which can serve as a reservoir for the transfer of antibiotic resistance genes (ARGs) to pathogenic bacteria [[4], [5], [6]]. These ARGs can be found on mobile genetic elements (MGEs), such as plasmids and transposons, facilitating their spread between different bacteria, including those that cause diseases in humans [7]. Horizontal gene transfer (HGT) of MGEs plays a vital role in the emergence and dissemination of AR in aquatic environments, including wastewater systems, where microbial density is high [1,8].

The emergence and mobilization of novel resistance genes in environmental bacteria, followed by their transfer to human pathogens, poses a significant risk to human health in relation to environmental AR [[3], [4], [5]]. Therefore, relying solely on clinical surveillance to prevent and control AMR is insufficient [3].

The One Health approach proposed by the WHO emphasizes the interconnectedness of human, animal, and environmental health in combating AR. It recognizes that all domains in which humans interact, including the environment, should be taken into account in the fight against antimicrobial resistance [9]. As part of this approach, the surveillance of AR in wastewater is considered a crucial component in monitoring and addressing AR in the environment. Additionally, the surveillance and analysis of antimicrobial resistance in wastewater treatment plants (WWTPs) offer significant advantages by providing a broader perspective on the presence of ARGs within a population [10]. This approach overcomes traditional surveillance limitations, such as limited sample sizes and data solely from hospitals [[4], [5], [6]]. It is worth noting that resistance data obtained from wastewater can serve as a reflection of the AR data observed in clinical settings [9].

Research on AR in wastewater, particularly in developing countries, has focused primarily on clinically relevant pathogens of fecal origin, such as *E. coli*, *K. pneumoniae*, *Enterobacter* species, and *Enterococcus* species [5]. While studying the presence of these pathogens in wastewater can offer insights into the distribution of AR and associated health risks, there is currently limited data available on the abundance and patterns of ARGs in wastewater. Surveillance of ARGs in wastewater has the potential to provide valuable information on the prevalence and trends of AR, as well as the emergence of resistant infections in society [5]. This information is particularly valuable in regions where clinical surveillance is limited due to inadequate infrastructure and resources [11].

It is important to note that various factors can contribute to the prevalence and spread of ARGs in wastewater [6]. The development of AR within communities is influenced by multiple factors, including the use of antibiotics in clinical and agricultural settings, economic and social factors, such as poor sanitation and hygiene practices, and inadequate surveillance systems [2,6]. Furthermore, several biotic and abiotic factors within wastewater systems can influence the dissemination of ARB and ARGs. These factors include the type of bacteria present, the concentration and types of antibiotics present, and the physicochemical characteristics of the wastewater, such as temperature, dissolved oxygen levels, and the presence of organic matter [2,6].

Given the importance of wastewater surveillance in the global action plan on antimicrobial resistance and the limited research conducted in developing countries, this study aimed to investigate the prevalence of the most important ARGs in wastewater. Previous studies have highlighted the presence of integrons, particularly class 1 integron-integrase gene (*intI*1), as efficient mechanisms for accumulating antimicrobial resistance genes, especially in gram-negative bacteria [7]. These integrons carry gene cassettes associated with AR, particularly in multidrug-resistant bacteria, and have been proposed as indicators of anthropogenic pollution in various environments [12]. Therefore, this study examined the abundance of five ARGs (*bla*_CTX-M_, *tet*W, *sul*1, *cml*A, *erm*B) and the *intI*1 gene in the effluent of two large-scale municipal WWTPs. Additionally, the relationship between effluent characteristics (BOD, COD, TSS, temperature, *E. coli*, and total coliforms) and the occurrence of ARGs and *intI*1 gene in the wastewater effluents was investigated. Furthermore, the bacterial hosts of the ARGs were identified for 381 important pathogens using the Comprehensive Antibiotic Resistance Database (CARD).

## 2 Materials and methods

### 2.1 Study sites and sampling

In the present study, we examined the effluent of two large-scale municipal WWTPs (32.7497 N, 51.7349 W and 32.6288 N, 51.7276 W) located in Isfahan, central part of Iran, covered distinct catchment regions and served a combined population of about two million of residents. In WWTP-A wastewater collects from both downtown and suburban regions, whereas WWTP-B specifically collects wastewater from the uptown region of Isfahan. It is worth noting that the socioeconomic conditions of the residents within the catchment area of WWTPB are generally superior to those residing within the catchment area of WWTP-A. Further characteristics of WWTPs are listed in Table 1.

**Table 1:** Characteristics of wastewater treatment plants.

WWTPs	Served population	Flow rate m^3^/d	Treatment process	Disinfection process	Final effluent receiving field
WWTP-A	1,149,000	229,785	Activated Sludge	Chlorination	Land application
WWTP-B	550,000	100,544	Activated Sludge	Chlorination (sometimes)	River

The surveillance was conducted over a period of one year from December 2018 to June 2019, during which a total of 50 grab samples were collected from the effluent of secondary treated wastewater. Of these samples, 13 were obtained from WWTP-A on a monthly basis, while 37 samples were collected from WWTP-B at 10-day intervals. All samples were collected in sterilized 1-L glass containers, transferred to the laboratory in an insulated box with cooling packs and analyzed for the physicochemical characteristics of wastewater, ARGs, *intI*1 and 16S rRNA genes.

### 2.2 Physicochemical and microbial analysis

Temperature and pH of wastewater samples were recorded at sampling site. Other physicochemical parameters including TSS (method 2540), BOD (method 5210, respirometric method), and COD (method 5220, colorimetric method), as well as microbial characteristics including total coliforms and *E. coli* (method 9221, multiple fermentation technique) were measured as described in *Standard Methods for the examination of water and wastewater* [13].

### 2.3 ARGs, *intI*1 and 16S rRNA genes detection

#### 2.3.1 DNA extraction

250 ml of the samples were centrifuged at 4020×*g* for 20 min, the supernatant was discarded, and the concentrates were collected. To extract genomic DNA, concentrates were treated with lysis buffer containing sodium dodecyl sulfate (SDS) and proteinase K, followed by incubation at 65 °C for 1 h. This was followed by a repeated freeze and thaw process using liquid nitrogen and boiling water, performed three times. DNA extraction and purification was then carried out using the Promega DNA Extraction kit (Promega Wizard Genomic DNA purification kit, Madison, WI) according to the manufacturer's manual. Extracted DNA samples were stored at −20 °C until further use.

### 2.4 Quantitative real-time PCR

Real-time PCR was used to quantify the abundance of ARGs (*bla*_CTX-M_, *tet*W, *sul*1, *cml*A, and *erm*B) and *intI*1 [14]. Quantification of 16S ribosomal RNA (16S rRNA) gene was also performed to determine the total bacterial population and normalize the abundance of ARGs in the samples based on the levels of 16S rRNA gene. The PCR primers and conditions for the amplification of ARGs, *intI*1 and 16S rRNA genes are listed in Table 2.

**Table 2:** Primers sequences used in the study.

Gene	Primer	Sequence (5' → 3′)	Amplified fragment (bp)	Annealing temperature (°C)	Reference
*tet*W	tet-W-F tet-W –R	GAGAGCCTGCTATATGCCAGC GGGCGTATCCACAATGTTAAC	168	64	[15]
*sul*1	sul1-F sul1-R	CGCACCGGAAACATCGCTGCAC TGAAGTTCCGCCGCAAGGCTCG	163	55.9	[15]
*erm*B	erm-B-F erm-B-R	AAAACTTACCCGCCATACCA TTTGGCGTGTTTCATTGCTT	193	60	[16]
*bla*_CTX-M_	CTX-M-F CTX-M-R	CGTCACGCTGTTGTTAGGAA CGCTCATCAGCACGATAAAG	156	60	[17]
*cml*A	cml-F cml-R	TAGTTGGCGGTACTCCCTTG GAATTGTGCTCGCTGTCGTA	137	60.5	[14]
*intI* 1	intI1-F intI1-R	GCCTTGATGTTACCCGAGAG GATCGGTCGAATGCGTGT	196	60	[18]
16S rRNA	16S–F 16S–R	GAAGATAATGACGGTATCTAAC ATTTCACACCTGACTGACTAT	139	58	[19]

The real-time PCR reactions were performed in a 15 μL volume and conducted in 48 well plates containing 7.5 μL of 2× SYBR Green (Ampliqon, Denmark), 0.2 μM of each primer, and 2 μL template DNA. All targeted genes were analyzed using an ABI prism equipment (Applied Biosystems, CA USA). The detailed protocol of the thermal cycle was as follows: initial denaturation at 95 °C for 10 min, followed by 40 amplification cycles (95 °C for 15 s and primer annealing at the selected annealing temperature for 45 s). Melting curve analysis was performed at the end of each qPCR run to confirm amplification specificity. All qPCR reactions were run in duplicates. Negative (molecular grade water) and positive (plasmid of studied genes) control were included in all runs. Furthermore, for confirmation of detected genes, sequencing of PCR products was conducted for several samples and analyzed using the BLAST database (http://www.ncbi.nlm.nih.gov/BLAST).

ARGs, *intI*1 gene, and 16S rRNA gene were quantified by plotting the quantification cycles (Cq) against a 10-fold serial dilution of the respective genes. This method allowed us to generate standard curves, following a previously described protocol [14]. The standard curves correlation coefficient (r) was greater than 0.99. Furthermore, CARD, a manually curated resource containing high quality reference data on the molecular basis of ARG was investigated to asses potential hosts of ARGs.

### 2.5 Statistical analysis

Statistical tests were conducted by using SPSS software (version 26). Initially, the normality of the data was evaluated. Comparisons of the average abundance of target genes in effluent samples of two WWTPs were done using non-parametric Mann-Whitney U tests. Spearman's correlation analysis was applied to the evaluation of the correlation between ARGs together with *intI*1 gene. A p-value of less than 0.05 was considered statistically significant. Further analysis and graphics were performed by R in software version 1.3.959. Specifically, Principal Component Analysis (PCA) was performed using ggfortify (https://cran.r-project.org/web/packages/ggfortify/index.html) and ggplot2 (https://cran.r-project.org/web/packages/ggplot2/index.html) packages. Heat maps and hierarchical clustering were performed using the stats package (https://www.rdocumentation.org/packages/stats/versions/3.6.2).

## 3 Results

### 3.1 Characteristics of wastewater effluents

Wastewater samples were analyzed to assess the influence of wastewater quality on the ARGs abundance (Table 3). The Mann-Whitney test showed no significant difference (p > 0.05) for effluent characteristics (BOD, COD, TSS, temperature, total coliforms and *E. coli*) between WWTP-A and WWTP-B.

**Table 3:** Physicochemical and microbial characteristics of wastewater effluents.

WWTPs	Total coliforms (MPN/100 ml)	*E. coli* (MPN/100 ml)	BOD (mg/L)	COD (mg/L)	TSS (mg/L)	Temperature (^o^C)
WWTP-A	2.33 × 10^7^	4.66 × 10^6^	42	109	54	21
WWTP-B	2.55 × 10^6^	7.48 × 10^5^	40	110	47	21

BOD: biological oxygen demand; COD: chemical oxygen demand; TSS: total suspended solids.

### 3.2 ARGs, *intI*1 and 16S rRNA genes abundance and distribution

**Fig. 1:**
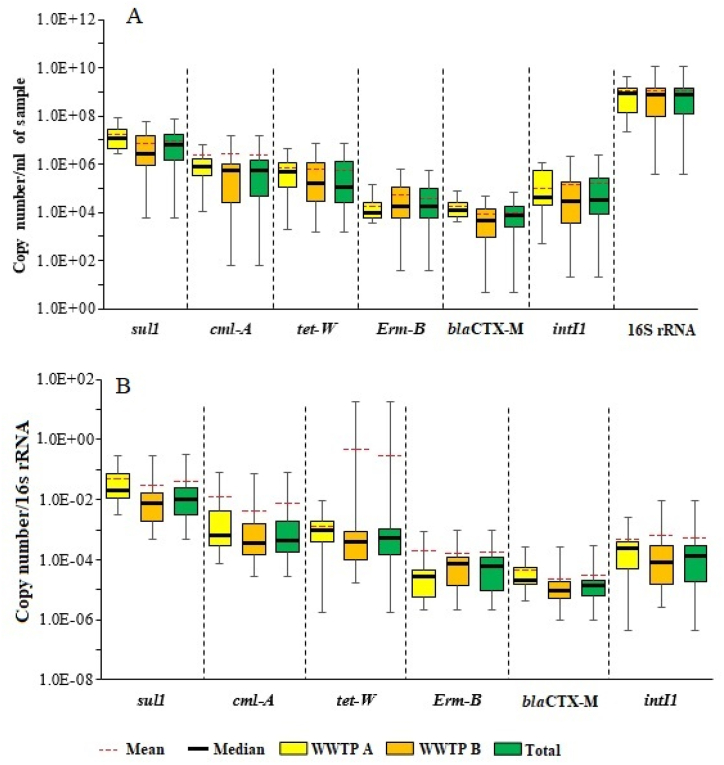
Abundance of ARGs, *intI*1 and 16S rRNA genes separated by different WWTPs: A) absolute abundance (gene copy number/ml of sample), B) normalized abundance (gene copy number/16S rRNA gene copy number).

Fig. 1A displays the absolute abundance and distribution patterns of ARGs, *intI*1, and 16S rRNA genes. Furthermore, in order to allow a more detailed comparison of the dynamics of the different genes, and minimize the differences in background bacterial abundances, numbers of all detected genes were normalized to 16S rRNA gene copy numbers (Fig. 1B). In general, all analyzed ARGs (*sul*1, *bla*_CTX-M_, *tet*W, *cml*A, and *erm*B) as well as *intI*1 and 16S rRNA genes were detected in all samples. Among the target ARGs, *sul*1 with a mean absolute value of 1.73 × 10^7^ copies mL^−1^ in WWTP-A and 8.95 × 10^6^ copies mL^−1^ in WWTP-B was the most abundant ARG. Furthermore, *bla*_CTX-M_ had the lowest abundance among the analyzed ARGs with a mean of 1.8 × 10^4^ copies mL^−1^ and 9.01 × 10^3^ copies mL^−1^ in WWTP-A and WWTP-B, respectively.

Specific attention was given to the abundance of *intI*1 gene as a proxy for anthropogenic pollution. *IntI*1 gene was found at approximately 10,000 times lower levels than 16S rRNA gene with a mean value of 2.15 × 10^5^ copies mL^−1^ in WWTP-A and 1.71 × 10^5^ copies mL^−1^ in WWTP-B. The abundance of *bla*_CTX-M_ and *erm*B was clearly below the levels of *intI*1 gene, whereas the rest of ARGs were found at counts generally exceeded *intI*1 gene abundance (Fig. 1A and B). Furthermore, 16S rRNA gene as a proxy for the bacterial load was found with a mean value of 9.93 × 10^8^ copies mL^−1^ in WWTP-A and 9.93 × 10^8^ copies mL^−1^ in WWTP-B.

ARGs and *intI*1 gene absolute and normalized abundance showed a similar pattern in WWTP-A and WWTP-B except for the normalized abundance of *tet*W in WWTP-B (Fig. 1B). However, as is evident in Fig. 1. ARGs and *intI*1 gene represent no significant changes between WWTP-A and WWTP-B. Furthermore, the Mann-Whitney test showed no significant difference (p > 0.05) for ARGs, *intI*1, and 16S rRNA genes between WWTP-A and WWTP-B. However, *sul*1 and *bla*_CTX-M_ were found at higher levels in WWTP-A.

The release rate of ARGs, *intI*1, and 16S rRNA genes through the effluents of WWTPs were calculated by multiplying the concentrations of genes by the average flow rate. As shown in Table 4, *sul*1 and *bla*_CTX-M_ had the highest and lowest release rates, respectively. The release rate of ARGs for WWTP-A was in the range of 4.1 × 10^15^ to 3.97 × 10^18^ copies d^−1^ and for WWTP-B was in the range of 9.06 × 10^14^ to 8.99 × 10^17^ copies d^−1^, respectively.

**Table 4:** Release rate of ARGs, *intI*1 and 16S rRNA genes through the effluents of WWTPs (copies d^−1^).

	*sul*1	*erm*B	*bla*_CTX__-M_	*cml*A	*tet*W	*intI*1	16S rRNA
WWTP-A	3.97 × 10^18^	4.9 × 10^15^	4.1 × 10^15^	2.86 × 10^17^	1.86 × 10^17^	4.93 × 10^16^	2.28 × 10^20^
WWTP-B	8.99 × 10^17^	6.84 × 10^15^	9.06 × 10^14^	1.43 × 10^17^	7.7 × 10^16^	1.72 × 10^16^	1.2 × 10^20^

### 3.3 Correlations between the abundance of ARGs, *intI*1 and 16S rRNA genes

**Fig. 2:**
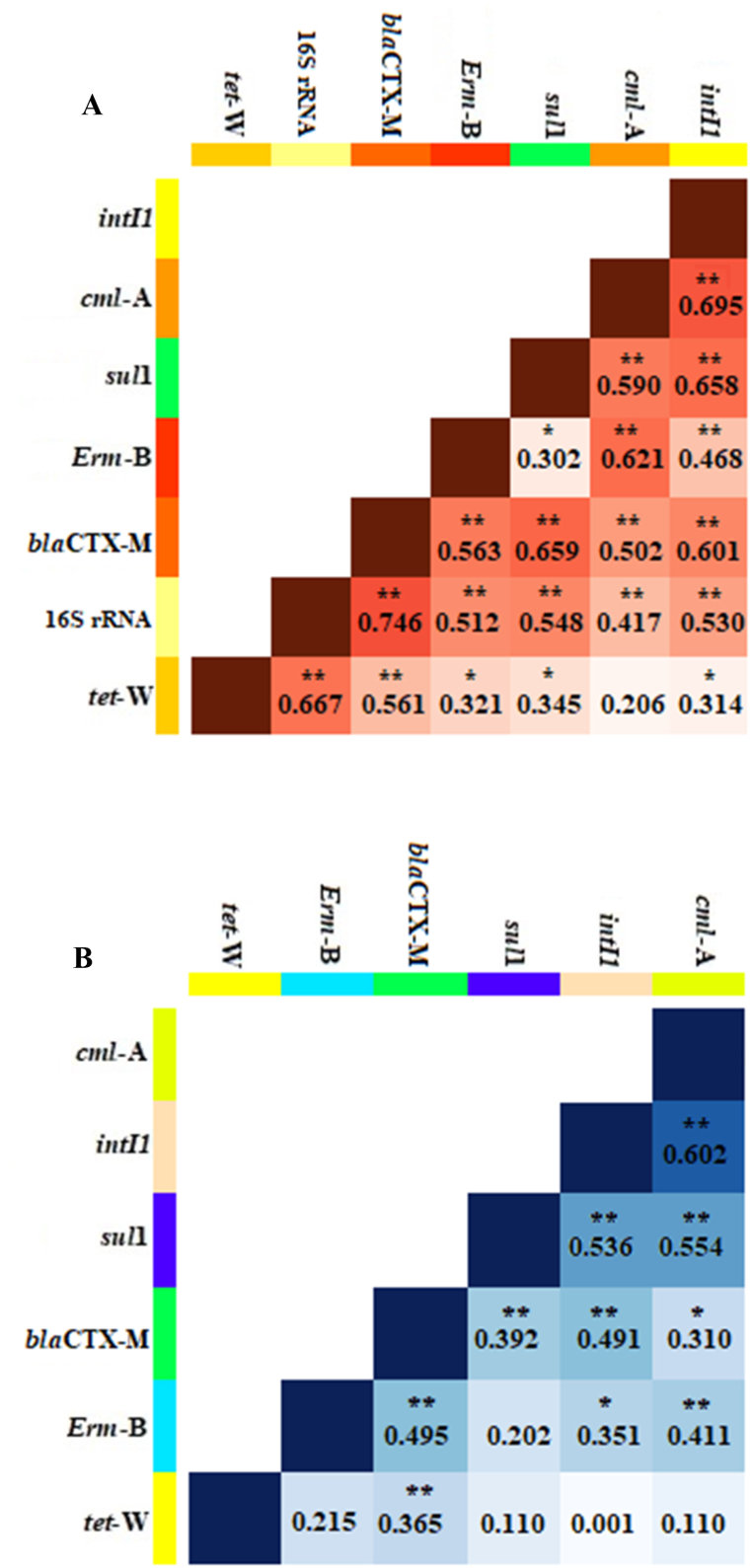
Heat map of Spearman's correlation among ARGs and *intI*1 gene: A) absolute abundance, B) normalized abundance. Asterisks indicate significant correlations (*, p < 0.05; **, p < 0.01).

The correlation between the absolute abundance of ARGs, *intI*1, and 16S rRNA genes is presented in Fig. 2A. For the absolute abundance, positive correlation was observed between all detected genes except *cml*-A and *tet*W which were not correlated together. Furthermore, *intI*1 gene exhibited a correlation with all ARGs and a high correlation was observed with the gene classes conferring resistance to chloramphenicol (*cml*A), sulfonamides (*sul*1), and β-lactamase (*bla*_CTX-M_). However, the normalized abundance of ARGs and *intI*1 gene exhibited a lower correlation (Fig. 2B).

### 3.4 Diverse and pathogenicity of ARG hosts

The prevalence of detected ARGs among the sequenced genomes, plasmids, and whole-genome shotgun assemblies of 381 important pathogens available in the CARD was investigated to asses potential hosts of ARGs. Based on the CARD, we extracted the four most prevalent target pathogens as presented in Supplementary data (Table S1). Our search results showed that the Enterobacteriaceae, which was found to carry the most diverse set of ARGs potentially are of great concern.

### 3.5 Relationship between normalized ARGs and *intI*1 gene abundances and effluent characteristics

**Fig. 3:**
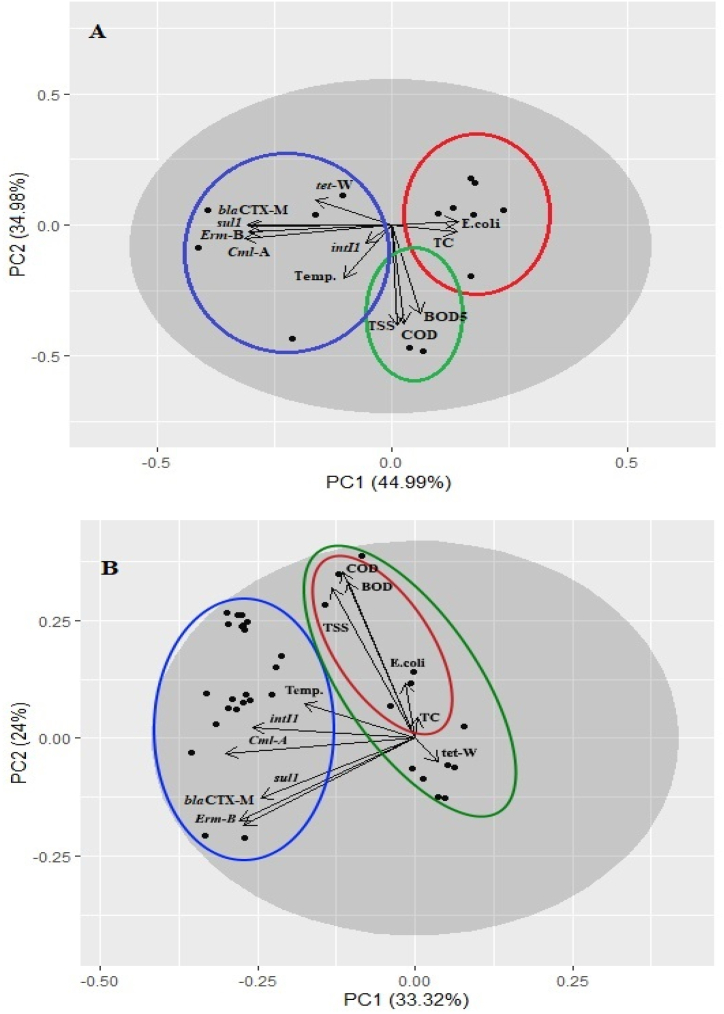
Evaluation of the effect of effluent characteristics on the ARGs and *intI*1 gene normalized abundance in A) WWTP-A, and B) WWTP-B. (COD: chemical oxygen demand; BOD: biological oxygen demand; TSS: total suspended solids; TC: total coliforms; Temp: effluent temperature).

PCA was performed for each WWTPs to examine the effect of effluent characteristics (BOD, COD, TSS, temperature, total coliforms and *E. coli*) on normalized ARGs and *intI*1 gene abundance. In both WWTPs, a total of nearly 79 % variation of the ARGs could be explained by the two axes of PCA (Fig. 3A and B).

In the WWTP-A, three main clusters based on effluent characteristics were evident, which were separated by *E. coli* and total coliforms (red cluster), BOD, COD, and TSS (green cluster), and effluent temperature (blue cluster). All ARGs and *intI*1 gene values were classified in blue cluster therefore, their direction was similar to the effluent temperature, but in the opposite direction of *E. coli* and total coliforms. However, based on the PCA the effect of *E. coli* and total coliforms on the abundance of ARGs is negligible and could not be considered. Whereas, BOD, COD, and TSS showed no significant effect on ARGs normalized abundance in WWTP-A.

In WWTP-B, two main clusters as well as one sub-cluster, were evident which were separated by *tet*W (green cluster) and effluent temperature (blue cluster) (Fig. 3B). *E. coli*, total coliforms, BOD, COD, and TSS (red cluster) were categorized as sub-cluster for *tet*W. As the WWTP-A all other ARGs and *intI*1 gene values were classified in the blue cluster which their direction was similar to the effluent temperature and its effect on ARGs and *intI*1 gene normalized abundance was decreasing. Whereas as shown in Fig. 3B, *E. coli*, total coliforms, BOD, COD, and TSS showed no significant effect on ARGs normalized abundance in WWTP-B. Furthermore, in both WWTPs, it seems that the abundance of *intI*1 gene was relatively correlated with BOD, COD, and TSS concentration.

## 4 Discussion

### 4.1 Distribution and abundance of ARGs, *intI*1 and 16S rRNA genes

The substantial presence of diverse ARGs in the effluent of WWTPs emphasizes that although WWTPs may effectively reduce the abundance of most ARGs, a wide range of ARGs still persist in the effluent. This persistence makes them readily available to environmental bacteria, including pathogenic strains. Consequently, these ARGs, along with ARBs, are discharged into the surrounding environment, leading to alterations in the diversity and distribution of ARGs within the ecosystem. This poses risks to both local humans and animals [1,14].

In the present study, the mean value of 16S rRNA gene copies (1.13 × 10^9^ copies mL^−1^) was consistent with previously reported values in 12 activated sludge treatment plant effluents in Poland [20]. However, Lapara et al. (2011) and Quach et al. (2018) reported significantly lower concentrations of 16S rRNA gene copies (5.4 × 10^6^ copies mL^−1^) in the effluent of tertiary-treated municipal wastewater. This suggests that the type of treatment technology employed is an important factor influencing the abundance of 16S rRNA gene, which serve as a proxy for bacterial load in WWTPs effluents [[21], [22]]. The abundance of *intI*1 gene was 1.82 × 10^5^ copies mL^−1^ which is similar to the findings reported by Niestępski et al. (2020) for the effluent of 12 activated sludge WWTPs [20].

Quantitative PCR analysis revealed the presence of all ARGs in 100 % of the samples. This finding is consistent with previous studies that have reported the presence of ARGs in a wide number of wastewater samples [8].

The absolute abundance of ARGs showed a similar distribution pattern in both WWTPs. Among the ARGs, *Sul*1, which is located on the 30-CS region of the classic class 1 integrons, exhibited the highest absolute abundance (1.11 × 10^7^ copies mL^−1^) in both WWTPs. This value was approximately one log higher than the second most abundant ARG, *cml-*A (Fig. 1A and B). Furthermore, the release rate of ARGs ranged from 9.06 × 10^14^ to 8.99 × 10^17^ copies d^−1^. Similar findings were reported by Munir et al. (2011) in the effluents of five WWTPs in the United States [15].

Consistent with our findings, a global study conducted across 60 countries showed that wastewater samples from Asian and African regions had a higher proportion of sulfonamides and phenicols resistance genes, while samples from Europe and North America had a higher relative proportion of genes conferring resistance to macrolides [6]. Sulfonamides are commonly used antibiotics in both human and veterinary medicine [23]. Ben et al. (2017) reported the abundance of *sul*1 in ten different WWTPs in China with a mean value of 1.1 × 10^6^ copies mL^−1^ [8]. Furthermore, Mao et al. (2015) detected sulfonamide resistance genes (*sul*1 and *sul*2) with an average abundance of 6.7 ± 7.2 × 10^5^ copies mL^−1^ as the most prevalent ARG in the effluent of two activated sludge WWTPs [24]. The high frequency of *sul*1 gene may be attributed to the high solubility of sulfonamides and their extensive historical use in medicine [25]. Furthermore, Makowska et al. (2016) found that the reduction of *sul* gene during treatment in the activated sludge system is less than one order of magnitude, indicating that the abundance of *sul* genes remains relatively stable throughout the treatment process [26].

Although no significant difference (p > 0.05) was observed in the distribution of ARGs and *intI*1 gene abundance between WWTP-A and WWTP-B, the abundance of ARGs and *intI*1 gene in WWTP-A was relatively higher in WWTP-A compared to WWTP-B (Fig. 1A and B). Due to the complexity of wastewater, the ARG abundance in WWTPs may be related to a variety of factors, such as antibiotic residues, environmental variables, effluent characteristics, as well as socioeconomic and health factors. Therefore, it is necessary to combine the multiple factors to evaluate the occurrence and fate of total ARGs in WWTPs [1]. Recent researches by Collignon et al. (2018) and Hendriksen et al. (2019) prove that while antibiotic consumption is often considered to be one of the most important factors driving resistance, the differences in AR between WWTPs could be best described by socioeconomic factors. Both studies found that factors related to sanitation and hygiene are significantly associated with AR abundance [6,27]. It should be noted that despite WWTP-B receiving a larger proportion of wastewater from healthcare facilities compared to WWTP-A, the abundance of ARGs and the *intI*1 gene was relatively higher in WWTP-A. This discrepancy could potentially be explained by socioeconomic factors and the presence of suburban small livestock in the catchment area of WWTP-A. It is also important to mention that residents in the catchment area of WWTP-B generally have better socioeconomic conditions compared to those in the catchment area of WWTP-A.

Furthermore, despite the higher concentrations of ARGs in healthcare wastewater systems compared to community, the discharge from healthcare facilities is estimated to represent only 1 % of the total influent volume of municipal wastewater [28]. Therefore, the contribution of healthcare facilities' wastewater is likely too small (about 1.29 % for WWTP-B) to significantly impact the overall concentration of these genes in WWTPs. Similar conclusions were recently drawn by Pallares-Vega et al. (2019) [28].

### 4.2 Correlations between the abundance of ARGs, *intI*1 and 16S rRNA genes

HGT plays a critical role in the widespread dissemination of AR. Within bacteria, ARGs can be acquired through the horizontal transfer of mobile MGEs like plasmids and transposons. Integrons are essential components in capturing and incorporating resistance gene cassettes. The most commonly identified integron types are class 1, 2, and 3, which frequently confer resistance to a wide range of antibiotics, including β-lactams, chloramphenicol, sulfonamides, aminoglycosides, spectinomycin, and others. The integrons are typically found within MGEs, which facilitate the dissemination of resistance genes within bacterial communities [7,29].

Therefore, the high prevalence of integrons, in particular *intI*1 as one of the most important elements in the development and transmission of ARGs among bacteria is alarming [20]. In this study, it was not surprising to find that all analyzed ARGs were correlated with the *intI*1 gene (Fig. 2). For instance, the *sul*1 gene, which was the most frequently detected ARG, showed a significant correlation with the *intI*1 gene. This correlation can be attributed to the fact that the *sul*1 gene is often located on the 3′-CS region of the classic class 1 integron-integrase gene [30]. Previous studies have also reported a link between human activities and the presence of *sul* genes in environmental samples [30,31]. It has been suggested that the presence of the *intI*1 gene in environmental samples is associated with anthropogenic activities [31]. It is important to note that the significant correlation between ARGs and the *intI*1 gene highlights the potential risk of ARG dissemination through HGT following the reuse of wastewater or the discharge of wastewater treatment plant effluents into the environment [8].

Although there was a correlation between the absolute and normalized abundance of almost all surveyed ARGs and the *intI*1 gene, a higher abundance of ARGs was detected compared to the *intI*1 gene except for *erm*B and *bla*_CTX-M_ (Fig. 1). This finding is consistent with a Mexican study on wastewater irrigation of soils, which also reported a higher abundance of ARGs compared to the *intI*1 gene [32]. It is therefore suggested that the higher abundance of ARGs may be attributed to their association with other MGEs or the presence of multiple copies of these genes in class 1 integrons [32].

Furthermore, all ARGs exhibited a strong correlation with the 16S rRNA gene, with Spearman's correlation coefficients ranging from 0.417 to 0.746 (Fig. 2A). This implies that the abundance of bacteria in the effluents could play a significant role in the horizontal transfer of ARGs [8]. Fig. 2A also demonstrates a strong correlation among almost all ARGs, indicating a genetic linkage driven by the co-occurrence and co-transfer of ARGs, which can contribute to multidrug resistance in bacteria. This result underscores the importance of studying the co-transfer of ARGs in receiving environments, which should receive more attention in future research [8].

### 4.3 Diversity and pathogenicity of ARG hosts

In order to gain insight into the potential hosts of ARGs and their associated health risks, we conducted a comprehensive investigation (Table S1). To assess this, we examined the prevalence of detected ARGs among the sequenced genomes, plasmids, and whole-genome shotgun assemblies of 381 important pathogens available in the CARD [33]. Our search results revealed that the Enterobacteriaceae carries the most diverse set of ARGs, which is of significant concern. It is worth noting that these microorganisms are capable of causing severe infections, including antibiotic-associated hemorrhagic colitis, urinary tract infections, gastroenteritis, and bacteremia, thereby posing a high health risk [[34], [35], [36]].

Analysis of the CARD revealed a strong relation between the Enterobacteriaceae family and gene classes associated with resistance to chloramphenicol (*cml*A), sulfonamides (*sul*1), and β-lactamase (*bla*_CTX-M_). This relation suggests the possibility of co-occurrence of multiple ARGs within this family, potentially leading to the presence of multidrug resistance. Among the Enterobacteriaceae family, there is a higher relative abundance of bacteria carrying ARGs in the genera *Klebsiella*, *Providencia*, *Proteus*, *Salmonella*, *Escherichia*, and *Shigella.* It is worth noting that Enterobacteriaceae are a natural part of the microbiota in the human intestinal tract and make up a significant portion of bacterial communities in wastewater. Therefore, the widespread occurrence of these bacterial populations in wastewater is to be expected. These findings are consistent with previous studies that have identified members of the Gamma-proteobacteria, such as Enterobacteriaceae, as the dominant ARB harboring diverse ARGs in wastewater treatment plants [1,37,38].

### 4.4 Relationship between normalized ARGs and *intI*1 gene abundances and effluent characteristics

PCA showed that in both WWTPs all ARGs and *intI*1 gene abundance were positively correlated with effluent temperature, but all other effluent characteristics (BOD, COD, TSS, total coliforms and *E. coli*) showed no significant correlation with ARGs abundance (Fig. 3). It has been reported that in biological treatment, the temperature as an influential parameter affects the performance of conventional activated sludge processes, which in turn could affect the microbial community, presence and abundance of ARB and ARGs [23,39,40]. In the present study, the effluent temperature was in the range of 10 °C–28 °C while the optimum temperature for biological reactions is typically between 15 °C and 35 °C [41]. Therefore, as is evident in Fig. 3, an increase in effluent temperature to the optimum level, could provide to the favorable conditions for bacterial growth and the subsequent proliferation of related ARGs [42]. Additionally, there is evidence suggesting that low temperatures can lead to a decrease in the acetylation enzyme gene, which is crucial for transcriptional regulation and plays a role in various biological functions, resulting in lower microbial growth [39]. However, there is some controversy regarding the effect of wastewater temperature on the profiles and abundance of ARB and ARGs. Table S2 illustrates a comparison for influence of physicochemical and microbial characteristics on the occurrence of antibiotic resistance and integrase genes for different studies.

Sabri et al. (2020) reported that winter is the optimal season for ARG proliferation [43], while Sun et al. (2016) found higher concentrations of ARGs and *intI*1 genes in summer and high-temperature conditions [40]. Consistent with our study, Sun et al. (2016) also observed a positive correlation between the temperature of mixed liquor and the distribution of ARGs in membrane bioreactors [40]. These discrepancies among studies may be attributed to factors such as the source and geographical location of the wastewater, patterns of antibiotic usage, variations in temperature and precipitation during the sampling period, hydrodynamic conditions, and wastewater disposal practices (Table S2) [43]. Additionally, the relationship between antibiotic consumption and the emergence and development of ARB and ARGs has been well documented [12]. Therefore, it is possible that the higher abundance of ARGs at higher temperatures is related to the increased solubility and bioavailability of persistent organic pollutants, such as antibiotics, at high temperatures [44].

It is important to note that although ARGs in WWTP-A were found to be in the opposite direction of total coliforms and *E. coli* (Fig. 3A), based on the PCA, the effect of these factors on the abundance of ARGs is negligible and could not be considered. This finding is supported by a study conducted by Ram and Kumar (2020) in India, which showed that ARGs abundance did not correlate with total coliforms and *E. coli*, accounting for only 11 % of the PCA data [45]. However, it is possible that ARGs are related to other members of Enterobacteriaceae family, such as *Salmonella, Klebsiella, Proteus, Providencia,* and *Shigella* or heterotrophs which make up the majority of the bacterial population in wastewater [46]. Novo and Manaia (2010) found that heterotrophs had lower reduction rates compared to enterobacteria, suggesting that heterotrophs may be more relevant vectors of AR in WWTPs than enterobacteria [47]. Additionally, Aali et al. (2014) reported a correlation between the abundance of *tet*W gene and HPC concentration in the final effluent of activated sludge in WWTPs, indicating that tetracycline-resistant bacteria contribute significantly to the HPC population (Table S2) [[46].

Osińska et al. (2019) reported that conventional WWTPs are ineffective in eliminating ARGs. In fact, biological WWTPs create an environment that is conducive to the spread of ARGs, and the diverse microorganisms present facilitate the transmission of genes through HGT [48].

As shown in Fig. 3, the concentrations of BOD, COD, and TSS in both WWTPs had no significant effect on the abundance of ARGs, which is consistent with findings from other studies [[8], [49]]. Cacace et al. (2019) found that the COD of WWTP effluent is not correlated with the concentration of ARG copies released from treatment facilities [49]. Similarly, Ben et al. (2017) reported that TSS concentrations were not significantly correlated with the concentrations of ARGs in WWTP effluent [8]. However, the abundance of the *intI*1 gene, which serves as a proxy for anthropogenic pollution, was affected by the concentrations of TSS, BOD, and COD. This finding is supported by Zieliński et al. (2021), who demonstrated statistically significant correlations between the abundance of integrase genes and the concentrations of COD and BOD [50].

Some studies suggest that various factors, such as system operation time, pH, and temperature, may influence the abundance of certain ARGs in WWTP effluent [23,40]. However, others have reported that wastewater characteristics do not have a significant correlation with the abundance of ARGs (Table S2) [[8], [51]]. Nonetheless, it is important to note that wastewater treatment conditions may have different implications for different antibiotic resistance groups [23]. Although the results of this study indicate that almost all examined ARGs exhibited similar behavior in response to the studied effluent characteristics, it is likely that other factors, beyond BOD, COD, and TSS, may affect the abundance of ARGs. Therefore, further research is needed to better understand these factors.

## 5 Conclusion

In this study, we analyzed the presence and abundance of ARGs in wastewater effluents as an important component of the One Health approach. Wastewater surveillance showed a relatively high detection of ARGs conferring resistance to various antibiotic classes, with the highest detection observed for *Sul*1. We found a significant correlation between ARGs and *intI*1 gene, indicating the risk of ARG dissemination in the environment through HGT following the discharge or reuse of WWTP effluents. Our results showed that in both WWTPs all ARGs and *intI*1 gene abundance were positively correlated with effluent temperature. However, no significant correlation was observed between ARGs and *intI*1 gene abundance and other effluent characteristics (BOD, COD, TSS, total coliforms, *E. coli*). Nevertheless, the abundance of *intI*1 gene, serving as a proxy for anthropogenic pollution, was affected by TSS, BOD, and COD concentrations. Furthermore, the analysis of potential hosts of ARGs by CARD indicated the potential presence of multidrug-resistant Enterobacteriaceae. However, further research is needed to understand the impact of other factors such as environmental variables and wastewater characteristics on ARGs abundance.

## Data availability

The data will be made available on request.

## Funding

This research was supported by the Vice Chancellery for Research at the Isfahan University of Medical Sciences (Grant No. 1402285).

## CRediT authorship contribution statement

**Zahra Shamsizadeh:** Writing – review & editing, Writing – original draft, Validation, Methodology, Investigation, Data curation. **Mahnaz Nikaeen:** Writing – review & editing, Writing – original draft, Supervision, Conceptualization. **Farzaneh Mohammadi:** Writing – review & editing, Software, Formal analysis. **Marzieh Farhadkhani:** Writing – review & editing, Writing – original draft, Methodology, Investigation. **Mehdi Mokhtari:** Writing – review & editing, Writing – original draft, Resources. **Mohammad Hassan Ehrampoush:** Writing – review & editing, Supervision, Resources.

## Declaration of competing interest

The authors of the manuscript " Wastewater surveillance of antibiotic resistance and class 1 integron-integrase genes: potential impact of wastewater characteristics on genes profile " declare that they have no conflict of interest.
