# Supplementary material for: Wastewater surveillance of antibiotic resistance and class 1 integron-integrase genes: Potential impact of wastewater characteristics on genes profile
Source: Heliyon. 2024 Apr 30;10(9):e29601. doi: 10.1016/j.heliyon.2024.e29601 (PMC11098788; doi:10.1016/j.heliyon.2024.e29601)
Supplement: Multimedia component 1 [file mmc1.docx]

Table S1: Diversity of ARG hosts

| Resistance gene | Target pathogen (Taxonomic Family) | | | |
| --- | --- | --- | --- | --- |
| *Sul* 1 | *Klebsiella huaxiensis*  (Enterobacteriaceae) | *Providencia heimbachae*  (Enterobacteriaceae) | *Proteus mirabilis*  (Enterobacteriaceae) | *Laribacter hongkongensis* [(Betaproteobacteria](https://en.wikipedia.org/wiki/Betaproteobacteria)) |
| *Cml*-A | *Proteus mirabilis*  (Enterobacteriaceae) | *Salmonella enterica*  (Enterobacteriaceae) | *Escherichia fergusonii*  (Enterobacteriaceae) | *Shigella flexneri*  (Enterobacteriaceae) |
| *Tet*- W | *Bifidobacterium thermophilum*  ([Bifidobacteriaceae](https://www.ncbi.nlm.nih.gov/datasets/taxonomy/31953)) | *Christensenella minuta*  ([Christensenellaceae](https://www.ncbi.nlm.nih.gov/datasets/taxonomy/990719)) | *Dysosmobacter welbionis*  ([Oscillospiraceae](https://www.ncbi.nlm.nih.gov/datasets/taxonomy/216572)) | *Enterocloster clostridioformis*  ([Lachnospiraceae](https://www.ncbi.nlm.nih.gov/datasets/taxonomy/186803)) |
| *Erm*- B | *Butyricimonas faecalis*  ([Odoribacteraceae](https://www.ncbi.nlm.nih.gov/datasets/taxonomy/1853231)) | *Eubacterium maltosivorans*  ([Eubacteriaceae](https://www.ncbi.nlm.nih.gov/datasets/taxonomy/186806)) | *Jeotgalibaca arthritidis*  ([Carnobacteriaceae](https://www.ncbi.nlm.nih.gov/datasets/taxonomy/186828)) | *Streptococcus*  ([Streptococcaceae](https://www.ncbi.nlm.nih.gov/datasets/taxonomy/1300)) |
| *bla*_CTX-M-32_ | *Escherichia coli*  (Enterobacteriaceae) | *Escherichia marmotae*  (Enterobacteriaceae) |  |  |

Table S2: The influence of physicochemical and microbial characteristics on the occurrence of antibiotic resistance and integrase genes (A review of different studies)

| WWTP system/Source | Physicochemical/ microbial parameter | Target gene | Results | Reference |
| --- | --- | --- | --- | --- |
| Municipal WWTP effluent | Temperature, pH, TSS, BOD, and COD, as well as total coliforms and *E. coli* | *bla*CTX-M, *tet*W, *sul*1, *cml*A, *erm*B, *intI*1, 16S  rRNA | - All ARGs and *intI*1 gene abundance were positively correlated with effluent temperature, but all other effluent characteristics (BOD, COD, TSS, *E. coli*, total coliforms) showed no significant correlation with ARGs abundance. | This study |
| Horizontal subsurface flow constructed wetland | NO_3_, NO_2_, NH_4_, Ntotal, BOD_7_, TOC, pH, Temperature | *tet*B, *tet*M, *erm*B, *amp*C, *sul*1, and *qnr*S | - The ARG concentrations in the effluent were affected by system operation parameters, especially system maturity and temperature. | [1] |
| Membrane bioreactors for municipal wastewater treatment | Chemical oxygen demand (COD), TN, NH_4_^+^-N, TP, Temperature, Conductivity | ATP-binding cassette (ABC), small multidrug resistance (SMR), major facilitator super family (MFS), Multidrug toxic compound extrusion (MATE) and b-lactamase (Classes A–C). | - TN, TP and COD of influent, temperature and conductivity of mixed liquor were significant (P < 0.05) correlated to the multiple antibiotic resistance genes distribution in MBRs.  - Conductivity and temperature seemed to be the major factors that affected the antibiotic resistance genes of the four MBRs. | [2] |
| Municipal wastewater treatment plant with A_2_O-MBR system | NH_4_^+^-N**,** COD, TP, TN, TOC | *tet*G, *tet*W, *tet*X, *sul*1, and *intI*1 | - The median concentrations of five resistance genes within a season were all relatively higher in spring than they were in other seasons; then, the values decreased in the summer, and *tet*W, *tet*X, and *sul*1 reached their lowest concen tration in autumn.  - COD exhibited significant correlation with *tet*W, and *sul*1 in influent, respectively. | [3] |
| Oxidation ditch | pH, temperature, DO, COD, TP, NH_4_^+^, NO_3_^−^ | *erm*B, *sul*1, *sul*2, *tet*W, 16SrDNA, *intI*1 | - ARGs were positively correlated to some nutrients and negatively correlated to DO. - A seasonal effect was observed for the presence of almost all ARGs in the river, except *tet*W, but could not explain all variations for all ARGs tested. | [4] |
| Hospital and municipal wastewaters | Total coliforms | *Tet*W | - No significant correlation was found between the levels of tet(W) gene and total coliforms bacteria in either raw wastewater or final effluent. - Conventional wastewater treatment plants didn’t contribute in effective reduction of *tet*W gene and wastewater efflu- ents are a potential source for dissemination of *tet*W gene into the natural environment | [5] |
| 16 urban WWTPs in 10 different European countries | Number of hospital beds in the catchment, number of biological stages, COD effluent concentration, number of hospitals in the catchment, geographical latitude, plant size | *blaTEM, OXA-48, OXA-58, CTX-M-15, CTX-M-32, KPC-3, sul1, tetM, mcr-1, 16S rRNA, intI1* | - The only factor that shows a significant correlation with the absolute abundance of ARGs was the number of biological steps. Both | [6] |
| Municipal WWTP effluent | Total organic carbon (TOC), inorganic carbon (IC), total nitrogen (TN), COD, NH_4,_ NO_3_, NO_2_, PO_4_, phosphate-total (TP). | *bla*TEM*, bla*SHV*, bla*OXA*, Amp*C*, sul*1*, sul*2*, erm*B*, erm*C*, erm*F*, mef*A*, tet*A*, tet*M*, qep*A*, qnr*S*, aad*A9*, aac(6′)-Ib-cr, dfr*A1*, intI*1*, intI*2*, intI*3 | - The concentration of total organic carbon (TOC), concentration of NH_4_, TP and PO_4_ were correlated with the concentrations of many genes that determine resistance to different groups of antibiotics, with the strongest correlations determined for the genes blaSHV, mefA and aac (6′)-Ib-cr. - Concentrations of COD and BOD proved to be strongly correlated with the concentrations of ARGs, including blaOXA, blaSHV, sul2, mefA, aac(6′)-Ib-cr and tetA, and with the integrase genes. | [7] |

**References**

[1] M. Pei, B. Zhang, Y. He, J. Su, K. Gin, O. Lev, G. Shen, S. Hu, State of the art of tertiary treatment technologies for controlling antibiotic resistance in wastewater treatment plants, Environ. Int. 131 (2019) 105026. https://doi.org/10.1016/j.envint.2019.105026.

[2] Y. Sun, Y. xiao Shen, P. Liang, J. Zhou, Y. Yang, X. Huang, Multiple antibiotic resistance genes distribution in ten large-scale membrane bioreactors for municipal wastewater treatment, Bioresour. Technol. 222 (2016) 100–106. https://doi.org/10.1016/j.biortech.2016.09.117.

[3] J. Du, J. Geng, H. Ren, L. Ding, K. Xu, Y. Zhang, Variation of antibiotic resistance genes in municipal wastewater treatment plant with A2O-MBR system, Environ. Sci. Pollut. Res. 22 (2015) 3715–3726. https://doi.org/10.1007/s11356-014-3552-x.

[4] N.A. Sabri, H. Schmitt, B. Van Der Zaan, H.W. Gerritsen, T. Zuidema, H.H.M. Rijnaarts, A.A.M. Langenhoff, Prevalence of antibiotics and antibiotic resistance genes in a wastewater effluent-receiving river in the Netherlands, J. Environ. Chem. Eng. 8 (2020) 102245. https://doi.org/10.1016/j.jece.2018.03.004.

[5] R. Aali, M. Nikaeen, H. Khanahmad, Z. Hejazi, M. Kazemi, A. Hassanzadeh, Occurrence of tetracycline resistant bacteria and resistance gene (fetW) in hospital and municipal wastewaters, Fresenius Environ. Bull. 23 (2014) 2560–2566.

[6] D. Cacace, D. Fatta-Kassinos, C.M. Manaia, E. Cytryn, N. Kreuzinger, L. Rizzo, P. Karaolia, T. Schwartz, J. Alexander, C. Merlin, H. Garelick, H. Schmitt, D. de Vries, C.U. Schwermer, S. Meric, C.B. Ozkal, M.N. Pons, D. Kneis, T.U. Berendonk, Antibiotic resistance genes in treated wastewater and in the receiving water bodies: A pan-European survey of urban settings, Water Res. 162 (2019) 320–330. https://doi.org/10.1016/j.watres.2019.06.039.

[7] W. Zieliński, E. Korzeniewska, M. Harnisz, J. Drzymała, E. Felis, S. Bajkacz, Wastewater treatment plants as a reservoir of integrase and antibiotic resistance genes – An epidemiological threat to workers and environment, Environ. Int. 156 (2021). https://doi.org/10.1016/j.envint.2021.106641.
